# Atopic Dermatitis Beyond Cutaneous Inflammation: The Interplay Between Progesterone, Testosterone, Gut Microbiota, and Immune Dysregulation

**DOI:** 10.3390/microorganisms14071565

**Published:** 2026-07-17

**Authors:** Patricia Guevara-Ramírez, Elius Paz-Cruz, Viviana A. Ruiz-Pozo, Santiago Cadena-Ullauri, Rafael Tamayo-Trujillo, Andrés Cadena, Nicole Hannah Jaramillo, Luci Hidalgo Melo, Ana Karina Zambrano

**Affiliations:** Universidad UTE, Facultad de Ciencias de la Salud Eugenio Espejo, Centro de Investigación Genética y Genómica, Quito 170129, Ecuador

**Keywords:** atopic dermatitis, testosterone, progesterone, microbiota, health

## Abstract

Atopic dermatitis (AD) is a chronic inflammatory skin disorder characterized by epithelial barrier dysfunction, immune dysregulation, and marked clinical heterogeneity. Although sex hormones and gut microbiota have independently been implicated in AD, their potential interactions remain incompletely understood. This narrative review integrates evidence from clinical, experimental, and preclinical studies examining the interplay among progesterone, testosterone, gut microbiota, and AD, with emphasis on immune regulation, epithelial barrier function, and gut–skin communication. Overall, the available evidence suggests that progesterone is predominantly associated with type 2 immune responses, alterations in epithelial barrier homeostasis, and context-dependent microbial remodeling. In contrast, physiological testosterone is generally associated with immunoregulatory effects and microbial profiles enriched in short-chain fatty acid-producing bacteria, whereas local androgen metabolism may contribute to skin barrier dysfunction. Current evidence also supports a bidirectional relationship between gut microbiota and sex hormones, whereby hormonal fluctuations influence microbial composition while microbial metabolism may modify steroid hormone bioavailability. Collectively, these findings support an integrated endocrine–microbial–immune framework that may contribute to AD beyond cutaneous inflammation. Although direct evidence simultaneously evaluating these components remains limited, this framework may help explain sex-specific differences in disease susceptibility, severity, and clinical course while guiding future mechanistic studies of the hormone–gut microbiota–skin axis.

## 1. Introduction

Atopic dermatitis (AD) is a chronic inflammatory skin disease characterized by recurrent eczema, intense pruritus, epidermal barrier dysfunction, and immune dysregulation. Although AD primarily manifests in the skin, it is increasingly recognized as a systemic disorder involving multiple biological pathways. Current evidence suggests that disease development and progression result from the interaction of genetic susceptibility, immune alterations, environmental exposures, hormonal factors, and microbial communities [1].

AD is one of the most common chronic inflammatory skin diseases worldwide, affecting over 200 million people [2]. Although often considered a childhood disorder, AD disease frequently persists into adulthood. The overall prevalence is estimated to be between 13% and 15% in children and between 3% and 10% in adults; however, in certain populations and cohorts, rates can be as high as 15–20% and 17%, respectively [3,4]. Geographic and population differences in the prevalence of AD suggest that environmental, lifestyle, and host-related factors contribute substantially to disease susceptibility and severity [5]. Environmental exposure, dietary habits, psychosocial stress, and alterations in microbial diversity have been proposed to contribute to immune dysregulation and epithelial barrier dysfunction in susceptible individuals [3,4,6].

Sex hormones are increasingly recognized as important regulators of physiological processes beyond reproduction. In addition to their endocrine functions, progesterone and testosterone have been implicated in the regulation of epithelial homeostasis, immune responses, and inflammatory signaling in multiple tissues, including the skin and gastrointestinal tract [7,8]. Both the skin and intestinal epithelium express steroid hormone receptors and can respond to hormonal fluctuations through changes in barrier integrity, mucus production, and immune activity [6,9]. Clinical observations have also reported variations in AD symptoms during periods of hormonal change, such as puberty, the menstrual cycle, pregnancy, the postpartum period, and menopause, suggesting a potential relationship between hormonal status and disease activity [9].

In parallel, growing evidence supports the involvement of the gut–skin axis in inflammatory skin disorders, including AD, psoriasis, and rosacea. The gut–skin axis refers to the bidirectional communication between intestinal microbial communities, immune regulation, metabolic signaling, and skin homeostasis [10]. Alterations in gut microbial composition have been associated with changes in epithelial barrier function, immune responses, and increased susceptibility to inflammatory skin disorders, highlighting the potential role of intestinal dysbiosis in AD pathogenesis.

A bidirectional relationship between sex hormones and the gut microbiota has also been increasingly recognized [11]. Hormonal fluctuations can influence microbial composition and metabolic activity, whereas intestinal microorganisms may participate in steroid metabolism and hormone transformation, potentially affecting hormone availability within the host [12]. In this context, the gut microbiota has emerged as a potential mediator linking endocrine regulation, immune function, and epithelial homeostasis [13,14].

Given the broad and complex role of sex hormones in immune regulation and host–microbiota interactions, this review focuses specifically on progesterone and testosterone as representative hormonal pathways through which endocrine signaling may influence microbial composition, immune responses, and epithelial barrier function. This focused approach was adopted to provide a more detailed examination of endocrine–microbial interactions potentially relevant to atopic dermatitis.

Although sex hormones, gut microbiota, and immune dysregulation have each been associated with AD, these factors are frequently investigated independently. Consequently, the interactions among endocrine signaling, microbial metabolism, and immune responses in AD remain incompletely understood. Therefore, this review aims to summarize current evidence regarding the relationship between sex hormones, gut microbiota, and AD, with particular emphasis on immune regulation, epithelial barrier dysfunction, and the gut–skin axis.

## 2. Study Selection Criteria

This narrative review was based on a comprehensive literature search conducted in PubMed, Scopus, and Google Scholar. The search strategy combined MeSH terms and keywords related to “atopic dermatitis”, “gut microbiota”, “skin microbiota”, “gut–skin axis”, “sex hormones”, “progesterone”, “testosterone”, “steroid metabolism”, “immune dysregulation”, and “epithelial barrier”.

No publication date restrictions were applied. However, most of the available evidence was published from 2019 onwards, reflecting the growing interest in host–microbiome interactions and microbial endocrinology. Earlier studies were included when they provided relevant mechanistic or foundational evidence.

The review considered clinical, epidemiological, experimental, and mechanistic studies addressing the role of sex hormones in atopic dermatitis, alterations in gut and skin microbiota, and hormone–microbiota interactions involved in immune regulation, epithelial barrier function, and microbial steroid metabolism.

Because direct evidence simultaneously linking sex hormones, microbiota, and atopic dermatitis remains limited, particularly for progesterone-related mechanisms, relevant studies from immunology, endocrinology, microbiology, and host–microbiome research were also included when they provided biologically plausible insights into the proposed endocrine–microbial–immune axis.

Accordingly, the available evidence was narratively integrated to organize biologically plausible relationships and identify testable hypotheses, rather than to establish a definitive causal pathway in AD. Because this study was not designed as a systematic review, no formal PRISMA-based study selection process, meta-analysis, or risk-of-bias assessment was performed.

## 3. Sex Hormones in Dermatitis

The skin functions as a dynamic endocrine organ, integrating hormonal, immunological, and environmental signals to maintain tissue homeostasis [15]. In atopic dermatitis (AD), disruption of this balance contributes to epidermal barrier dysfunction, immune dysregulation, and chronic inflammation. Within this context, sex hormones have been identified as important modulators of both cutaneous structure and immune responses, helping to explain part of the clinical heterogeneity observed in AD [15,16]. Clinical observations indicate that disease severity often fluctuates during periods of hormonal change, including puberty, the menstrual cycle, pregnancy, and menopause, suggesting a close relationship between endocrine signaling and AD activity [17,18,19].

Among sex hormones, progesterone has been primarily associated with the promotion of type 2 immune responses, a hallmark of AD pathophysiology [9,20,21]. Experimental and preclinical studies suggest that progesterone may promote type 2 immune responses by enhancing Th2 polarization and regulatory T-cell activity while suppressing Th1 and Th17 pathways. This immunological shift favors an inflammatory milieu associated with allergic responses and may contribute to disease persistence [9,22,23]. This effect has been proposed to involve the progesterone-inducible blocking factor (PIBF), a protein that amplifies IL-4 signaling and type 2 immune pathways [9,22,24,25]. Progesterone has also been associated with increased IgE production and enhanced expression of epithelial cytokines involved in allergic inflammation. However, the extent to which these mechanisms contribute to disease activity in human AD remains incompletely understood [22,24,26].

Beyond its immunological effects, progesterone may also influence epidermal barrier function. Experimental studies have shown delayed recovery of epidermal permeability following barrier disruption in the presence of elevated progesterone levels [27,28,29,30]. Consistent with these observations, clinical studies have reported increased transepidermal water loss during the luteal phase of the menstrual cycle, when circulating progesterone concentrations are highest [9,19,22]. Together, these findings suggest that progesterone may contribute to both immune activation and barrier dysfunction in susceptible individuals [31,32].

In contrast, testosterone and other androgens generally exhibit immunomodulatory properties that may be protective in AD [33]. Unlike progesterone, which promotes allergic inflammation, systemic testosterone generally exerts anti-inflammatory and immunomodulatory effects [32]. Experimental evidence suggests that testosterone can suppress the differentiation of Th2 lymphocytes, which are central drivers of AD, as well as Th1 and Th17 responses. In parallel, androgens may promote regulatory T cells through interactions with the *Foxp3* gene promoter, contributing to immune tolerance and attenuation of inflammatory responses [33,34,35,36]. This immunological profile suggests that reduced systemic testosterone may contribute to impaired regulation of inflammatory responses.

Testosterone may also influence the skin barrier. In contrast to estrogens, testosterone has been associated with reduced skin permeability, suggesting a potential role in maintaining epidermal barrier integrity [33]. Therefore, reduced systemic androgenic signaling could theoretically contribute to greater barrier vulnerability and disease worsening in AD [33]. In this context, disease severity has been linked to low serum testosterone levels in men, and adult male patients with AD have been reported to show significantly lower free testosterone levels than healthy subjects [9,37,38].

However, the role of testosterone in AD cannot be interpreted only from a systemic perspective. At the local level, AD skin may show increased androgen synthesis that negatively affects barrier function. In AD lesions, type 2 cytokines such as IL-4 and IL-13 activate *STAT6*, which binds directly to the promoter of the *HSD3B1* gene in sebaceous glands, markedly increasing its expression [33,39]. Increased HSD3B1 expression has been proposed to enhance local androgen synthesis and subsequent activation of *INSIG1*, a regulator of lipid metabolism. This pathway may reduce lipid synthesis and could contribute to the xerosis and barrier dysfunction in AD [33,39].

Overall, current evidence suggests a role for sex hormones as modulators of both immune and barrier-related pathways involved in AD (Table 1). Although the underlying mechanisms remain incompletely understood, hormonal signaling may contribute to interindividual differences in disease susceptibility, severity, and clinical course.

## 4. Hormones and Gut Microbiota Interactions

Sex hormones and the gut microbiota may interact through bidirectional mechanisms involving immune regulation, microbial metabolites, and local steroid transformation. Hormonal fluctuations can alter microbial composition and function, whereas gut microorganisms possess enzymatic pathways capable of transforming steroid substrates within the intestinal lumen. However, the extent to which these microbial activities affect circulating progesterone or testosterone concentrations in humans remains uncertain. In this section, we summarize current evidence on microbiota-mediated steroid transformation and the microbial patterns associated with progesterone and testosterone.

### 4.1. Microbiota-Mediated Steroid Metabolism and Intestinal Steroidogenesis

Current evidence suggests that interactions between the gut microbiota and sex hormones extend beyond compositional changes and involve microbial deconjugation and local transformation of steroid substrates [13,41,42]. One of the principal mechanisms described is bacterial β-glucuronidase activity during enterohepatic processing. After hepatic conjugation, steroid metabolites may reach the intestine through bile, where bacterial enzymes can deconjugate them and release free steroid compounds within the intestinal lumen [41,42]. Some of these compounds may subsequently be reabsorbed; however, the extent to which this process produces clinically meaningful changes in circulating progesterone or testosterone concentrations in humans remains uncertain. Therefore, enterohepatic deconjugation should be understood as a potential contributor to hormone bioavailability rather than as direct evidence of systemic endocrine regulation by the gut microbiota.

Beyond deconjugation, intestinal epithelial cells and resident microorganisms can participate in the local interconversion of steroid compounds [41,43,44]. Bacterial hydroxysteroid dehydrogenases, steroid reductases, and related enzymes have been identified across members of the phyla Firmicutes, Actinobacteria, and Proteobacteria, indicating that the gut microbiota possesses the metabolic capacity to modify steroid substrates within the intestinal lumen [41,42]. These reactions are most appropriately interpreted as changes in the local intestinal sterol pool rather than as direct evidence of systemic endocrine regulation.

Different bacterial taxa may participate in distinct steroid-transforming reactions depending on the enzymes they encode and the substrates available. *Gordonibacter pamelaeae*, *Eggerthella lenta*, and *Clostridium innocuum* have been associated with the transformation of progesterone-derived metabolites and neuroactive steroid-related compounds [13,45]. Similarly, *Clostridium scindens* can convert glucocorticoids into androgen precursors through side-chain cleavage reactions mediated by the steroid-17,20-desmolase encoded by the desA and desB genes [41,43]. Other taxa, including *Eggerthella lenta* and *Ruminococcus gnavus*, possess steroid-transforming enzymes involved in the interconversion of androgen-related metabolites [42]. However, the presence of these enzymatic pathways does not by itself demonstrate that bacterial activity produces clinically meaningful changes in circulating progesterone or testosterone concentrations.

Experimental studies suggest that changes in the gut microbiota may be associated with host androgen status. For example, administration of *Lactobacillus reuteri* was associated with restoration of testosterone levels in aged mice, indicating a potential microbiota-related effect in this animal model. In humans, increased abundance of taxa such as *Lachnoclostridium*, *Blautia*, and *Bergeyella* has been associated with lower circulating testosterone levels and metabolic dysfunction in men with type 2 diabetes [14]. However, these observational associations do not establish that the identified microorganisms directly modify circulating testosterone concentrations. Differences in metabolic status, inflammation, diet, medication use, and host endocrine function may also contribute to the observed relationships.

Beyond direct steroid transformation within the intestinal lumen, gut dysbiosis may also be indirectly associated with host endocrine function through inflammatory and metabolic pathways. Microbial products such as lipopolysaccharide can promote systemic inflammation and may interfere with steroidogenic signaling, whereas chronic inflammation can disrupt hypothalamic–pituitary–gonadal axis activity and contribute to reduced testosterone production [14,43]. These indirect host-mediated mechanisms should be distinguished from the local microbial transformation of steroid substrates.

Microbial steroid metabolism also includes reductive and oxidative reactions that can generate metabolites with biological properties distinct from those of their parent compounds. For example, the OsrABC pathway described in *Clostridium steroidoreducens* enables the reduction of natural and synthetic steroid substrates within experimental systems [45]. Similarly, bacterial enzymes with 5α- or 5β-reductase activity can produce steroid derivatives with altered androgenic, neuroactive, or immunological properties. These findings demonstrate the capacity of gut bacteria to modify the local intestinal sterol pool; however, they do not establish that these transformations produce clinically meaningful changes in circulating hormone concentrations in humans.

Collectively, these findings indicate that the gut microbiota possesses substantial enzymatic capacity to deconjugate and transform steroid compounds within the intestinal environment. These activities may influence local steroid availability and, potentially, enterohepatic processing. Nevertheless, most of the proposed systemic endocrine effects have been derived from in vitro experiments, animal models, or observational human studies. Their contribution to circulating progesterone and testosterone concentrations and their clinical relevance to AD, therefore, remain uncertain.

### 4.2. Progesterone and Microbiota Interactions

The relationship between progesterone and the gut microbiota remains incompletely understood and appears highly dependent on the physiological and inflammatory context. As summarized in Table 2, most available studies have focused on how progesterone influences microbial composition and function. During progesterone-dominant states, such as the luteal phase and pregnancy, microbial diversity often decreases, whereas genera including Lactobacillus and Bifidobacterium tend to expand [21,46]. Experimental studies further suggest that elevated progesterone levels may influence not only the abundance of individual taxa but also overall microbial community structure and beta diversity, indicating a potential role for progesterone in shaping microbial ecosystem organization [46,47].

Although *Lactobacillus* and *Bifidobacterium* were recurrently enriched across several progesterone-associated conditions, the biological significance of these changes appeared highly dependent on the surrounding physiological and inflammatory environment [21]. During pregnancy and hormone supplementation, expansion of these genera has been associated with pathways related to glycan metabolism, microbial metabolite production, epithelial homeostasis, and maternal immune tolerance [21,53]. However, inflammatory conditions such as pregnancy-induced immune thrombocytopenia have also been associated with increased *Lactobacillus* abundance accompanied by reduced microbial diversity and depletion of taxa commonly linked to intestinal homeostasis, including *Faecalibacterium*, *Blautia*, and *Parasutterella* [48]. At the same time, inflammation-associated genera such as *Helicobacter*, *Mucispirillum*, and certain *Bacteroides* species tend to expand under dysbiosis conditions [48,50]. These findings indicate that progesterone-associated microbial remodeling does not necessarily reflect a uniformly beneficial microbial state, but rather context-dependent ecological shifts shaped by both endocrine and inflammatory factors.

Several progesterone-associated microbial alterations involve bacterial genera linked to short-chain fatty acid (SCFA) metabolism, including *Roseburia*, *Lachnospira*, *Faecalibacterium*, and *Ruminococcus* [54]. Because SCFAs contribute to epithelial integrity, mucus production, and immune regulation, changes in these microbial communities may influence intestinal permeability and inflammatory signaling. In parallel, in vitro studies have demonstrated that progesterone can directly affect bacterial growth, adherence, and biofilm formation, suggesting that steroid hormones may exert ecological pressure on microbial communities independently of host immune responses [55,56]. Interestingly, progesterone and synthetic progesterone derivatives have also shown bactericidal activity against *Helicobacter pylori*, further suggesting that steroid hormones may directly influence microbial niche composition under specific conditions [51,55].

Compared with testosterone-associated microbiota research, studies specifically evaluating progesterone–gut microbiota interactions remain relatively limited and heterogeneous. Many investigations have been conducted in pregnancy-associated conditions, infertility models, inflammatory disorders, or non-intestinal microbial environments, including studies of the vaginal microbiota. Despite these limitations, the available evidence suggests that progesterone may contribute to context-dependent microbial and metabolic remodeling. However, the biological significance of these microbial alterations and their potential contribution to AD remain largely unexplored, highlighting the need for studies directly evaluating progesterone–microbiota interactions in this disease.

### 4.3. Testosterone–Microbiota Interactions

Testosterone has been associated with differences in the diversity, structure, and taxonomic composition of the gut microbiota. Although experimental designs, study populations, biological models, and sequencing methodologies differ across studies, the evidence summarized in Table 3 suggests a common trend linking elevated testosterone levels to a more diverse gut microbiota and a distinct taxonomic profile, characterized primarily by enrichment of Firmicutes-associated taxa and a relative reduction in taxa potentially associated with inflammation or dysbiosis [42,43,57,58].

One of the most consistently reported findings is the positive association between gut microbiota alpha diversity and endogenous testosterone levels [43,57,58]. Studies in healthy men have shown that higher plasma testosterone concentrations are associated with increased microbial richness and diversity, suggesting a more complex gut microbial ecosystem compared with individuals with medium or low testosterone levels [43,57].

Beyond this cross-sectional association, experimental and interventional evidence suggests that androgens, particularly testosterone and dihydrotestosterone, may contribute to the maturation and remodeling of the gut microbiome [43,57,58,68]. In prepubertal mice, androgen exposure has been associated with increased alpha diversity in fecal samples [68]. However, in transgender men undergoing testosterone treatment, early changes in alpha diversity may be limited or even temporarily reduced, while the most significant changes occur in beta diversity [57]. This suggests that, during the early stages of testosterone exposure, changes may occur mainly in the relative abundance of specific microbial taxa, rather than in the overall alpha diversity of the gut microbiota.

The effects of testosterone on the gut microbiota also appear to vary under physiological stress. For instance, during severe energy deficit, testosterone administration does not seem to fully prevent broad alterations in microbial diversity [58]. Nevertheless, some studies suggest that it may help preserve specific functional niches, particularly those related to intestinal barrier integrity and adaptive microbial metabolism [42,69]. Overall, these findings indicate that androgen–microbiota interactions are context-dependent and may be influenced by baseline hormonal status, physiological stress, and the duration of androgen exposure.

At the taxonomic level, several studies have reported relatively consistent patterns in the composition of the gut microbiota associated with elevated testosterone. *Ruminococcus*, a Firmicutes genus, has been frequently linked to higher circulating testosterone and has shown one of the strongest positive correlations with plasma testosterone concentrations in men. This association may be biologically relevant because *Ruminococcus species* are known to participate in the degradation of complex polysaccharides, the production of fermentation-derived metabolites, and the maintenance of an anaerobic intestinal environment [43].

At the phylum level, testosterone has been associated with a relative increase in Firmicutes. This pattern has been observed in both human studies and animal models, including Asian cohorts and studies in pigs [43,70]. Some studies have also reported a relative reduction in Bacteroidetes, potentially increasing the Firmicutes/Bacteroidetes ratio [43]. Although this ratio should be interpreted with caution, as it does not constitute a universal marker of health or dysbiosis, it may reflect an ecological reorganization of the gut microbiome under androgenic influence.

Other taxa positively associated with elevated androgenic profiles include *Dorea*, *Megamonas*, *Acinetobacter*, and members of the family Muribaculaceae [42,68]. Nevertheless, these findings appear less consistent than those reported for *Ruminococcus* and Firmicutes, and should therefore be interpreted as emerging associations rather than definitive markers of a testosterone-associated microbial profile [43].

In contrast, some taxa have shown inverse associations with testosterone levels. Among them, Bacteroidetes—and particularly the genus Bacteroides—have been negatively associated in some studies with higher circulating testosterone levels or testosterone-related metabolites [42,43]. Similarly, reductions in Proteobacteria and potentially pathogenic genera such as *Escherichia-Shigella* have been reported under conditions of increased androgenic exposure. This finding is relevant because an expansion of Proteobacteria is often interpreted as a potential indicator of ecological instability, intestinal inflammation, or dysbiosis [42,43].

Although several testosterone-associated microbial patterns have been identified, much of the available evidence remains observational. Therefore, further studies are required to determine the mechanisms and physiological relevance of these associations.

## 5. Discussion

In this review, we integrate three lines of evidence that have often been studied independently: sex hormones, the gut microbiota, and AD. Overall, the studies analyzed suggest that these systems may interact through interconnected endocrine, microbial, and immunological pathways that influence epithelial homeostasis, systemic inflammation, and immune regulation. In this context, the gut microbiota may represent an intermediary axis linking hormonal fluctuations with biological processes relevant to AD pathophysiology. Beyond taxonomic changes, findings from human and experimental studies suggest that sex hormones may influence microbial composition and metabolic activity, which could in turn affect immune responses and components of the gut–skin axis. However, many of these interactions have been inferred from indirect evidence, and the mechanisms through which they may contribute to AD pathophysiology remain incompletely understood. Taken together, these observations support an endocrine–microbial–immune model that should be viewed as a hypothesis-generating framework grounded in biological plausibility and supported primarily by indirect evidence, rather than as a confirmed mechanistic pathway in patients with AD.

To contextualize the role of progesterone and testosterone within this endocrine–microbial–immune framework, it is important to consider their steroidogenic origin. Both hormones belong to the network of cholesterol-derived steroids [71]. Steroidogenesis begins with the conversion of cholesterol into pregnenolone, a reaction catalyzed by *CYP11A1*. Pregnenolone can then be converted into progesterone by 3β-hydroxysteroid dehydrogenase (3β-HSD). Subsequently, testosterone synthesis can occur through two major steroidogenic pathways. The Δ4 pathway converts progesterone to 17α-hydroxyprogesterone, then to androstenedione, and finally to testosterone by 17β-hydroxysteroid dehydrogenase (17β-HSD). In the Δ5 pathway, pregnenolone is converted to 17α-hydroxypregnenolone and then to dehydroepiandrosterone (DHEA), which can be converted to androstenedione and testosterone [42,71].

Together, these pathways illustrate that progesterone plays an important role in steroidogenesis, acting both as an effector hormone and as a precursor for other steroidogenic pathways, including those leading to glucocorticoids, mineralocorticoids and androgens. However, an increase in serum progesterone does not necessarily mean a corresponding increase in testosterone, since this conversion requires specific enzymatic activities, especially 17α-hydroxylase, 17,20-lyase and 17β-HSD, which are expressed and active in a tissue-specific manner [42,71].

This tissue-specific requirement for steroid conversion is particularly relevant when analyzing the gut and skin. Unlike classical steroidogenic organs, such as the gonads, adrenal cortex, and placenta, the intestinal tract should not be regarded as a primary endocrine source of progesterone or testosterone. However, in the context of steroid signaling, it should not be considered a biologically inert tissue. The gut is exposed to circulating sex hormones, expresses hormone receptors, contains highly active immune cell populations, and supports a microbial ecosystem with its own metabolic capacity [42,43,58]. Thus, the gut represents a compartment in which systemic endocrine signals, microbial metabolism, barrier function, and mucosal immunity converge to shape biological processes potentially relevant to gut–skin communication.

In this context, one of the most consistent findings in AD-related hormonal research was a recurrent change in bacteria that produce short-chain fatty acids (SCFAs), particularly butyrate. *Faecalibacterium*, *Roseburia*, *Ruminococcus*, *Bifidobacterium* and members of the Lachnospiraceae family have often been associated with intestinal homeostasis, immune regulation and the maintenance of epithelial integrity. In several cohorts of patients with AD, a decrease in these taxa has been observed alongside the expansion of potentially pro-inflammatory bacteria, suggesting that reduced production of microbiota-derived metabolites may contribute to increased intestinal permeability and enhanced systemic inflammatory responses (Table 4) [43,72,73].

The importance of these alterations lies in the fact that butyrate regulates epithelial tight junctions, mucus production, energy consumption of colonocytes, and immune responses. In particular, a reduction in butyrate-producing bacteria could result in a decline of immunological tolerance. This would decrease regulatory signals mediated by regulatory T cells, leading to increased susceptibility to type 2 inflammatory responses, which are key hallmarks of AD [81,82,83]. Therefore, changes in these bacterial communities may represent an important point of functional convergence between sex hormones, the microbiota, and AD-related inflammation.

The patterns observed for testosterone and progesterone seem to be different within this framework. Profiles associated with physiological testosterone showed a relatively more consistent trend toward enrichment of bacteria linked to ecological stability and SCFA metabolism, such as *Ruminococcus*, *Roseburia* and members of the Lachnospiraceae family. Interestingly, a number of these taxa were also found to be reduced in cohorts with AD, suggesting that part of the protective effect attributed to testosterone could be indirectly mediated by the gut microbiota and its ability to preserve environments rich in immunoregulatory metabolites. In contrast, states associated with decreased testosterone or experimental castration were associated with decreased bacterial diversity and loss of communities associated with intestinal homeostasis [42,43,64].

These microbiota-related patterns are also consistent with the broader immunoregulatory role attributed to androgens. Therefore, it is plausible that some of the protective effects associated with physiological testosterone levels involve interactions between endocrine signaling and microbiota-associated immune pathways. Reduced testosterone levels could weaken these regulatory mechanisms and favor inflammatory pathways associated with AD. However, it is important to distinguish between the systemic effects of testosterone and its local metabolism in inflamed skin. Although circulating testosterone is involved in epithelial homeostasis, local androgen production in AD lesions may have different outcomes. In inflamed skin, type 2 cytokines (IL-4, IL-13) can induce sebaceous gland expression of *HSD3B1* and enhance local androgen synthesis. This local increase in androgen signaling can upregulate *INSIG1*, downregulate triglyceride synthesis and alter the lipid composition required for a competent skin barrier. Thus, systemic testosterone deficiency and local androgen excess in skin lesions should not be considered contradictory mechanisms but compartmentalized processes that can contribute to AD via different pathways [9,34,36,39].

Beyond the gut microbiota, increasing evidence indicates that cutaneous microbial communities also contribute to AD pathophysiology and interact with local hormonal signaling. AD-associated skin dysbiosis is characterized by increased colonization with Staphylococcus aureus together with reduced abundance of protective commensal microorganisms, including *Staphylococcus epidermidis*, *Corynebacterium* spp., and other coagulase-negative staphylococci [80,84]. This microbial imbalance has been associated with reduced microbial diversity, impaired colonization resistance, barrier dysfunction, and amplification of cutaneous inflammatory responses. In addition, studies comparing AD patients with healthy controls have consistently reported a negative correlation between *S. aureus* abundance and the presence of commensal skin bacteria, suggesting that loss of microbial competition may facilitate pathogen overgrowth and disease exacerbation [80,85].

Recent experimental findings further suggest that androgens may not only regulate host immune responses but also directly influence bacterial behavior at the skin surface. Testosterone and dihydrotestosterone have been shown to enhance *Staphylococcus aureus* virulence through activation of the accessory gene regulator (agr) quorum-sensing system, promoting the expression of cytotoxic factors involved in skin inflammation and tissue damage [86,87]. Furthermore, reduced local androgen production was associated with lower susceptibility to *S. aureus* infection, whereas topical testosterone increased bacterial pathogenicity and skin barrier disruption. Although these findings remain largely experimental, they suggest that local androgen signaling may influence host–microbe interactions within inflamed skin and warrant further investigation in AD-specific settings [86].

In contrast to the relatively consistent microbial patterns associated with physiological testosterone levels, progesterone-related microbial remodeling appeared substantially more heterogeneous and highly dependent on the endocrine and inflammatory environment. Rather than promoting a single characteristic microbial configuration, progesterone seems to induce dynamic ecological adaptations shaped by host physiology, immune activity, and hormonal fluctuations. These observations support the idea that progesterone may function primarily as an immunometabolic regulator capable of continuously reshaping microbial ecosystem organization under hormonally variable conditions [43,64].

In this context, the increase in genera such as Lactobacillus may represent adaptive responses to fluctuating hormonal environments rather than uniformly protective microbial changes. This interpretation is especially relevant because physiologically progesterone increases during the luteal phase and pregnancy, when it can affect intestinal function by decreasing motility, slowing gastrointestinal transit, modifying mucus secretion, modulating the epithelial barrier, and regulating mucosal immunity. These effects should not be considered pathological because they could be associated with compensated physiological adaptations. However, in susceptible individuals, particularly those with AD, the cycle-dependent increase in progesterone may exacerbate pre-existing alterations in barrier and type 2 immunity [88,89].

The immunological effects of progesterone may become particularly relevant in individuals already predisposed to type 2 inflammation and barrier dysfunction. In this context, progesterone-dependent microbial remodeling and immune modulation could amplify pre-existing inflammatory pathways associated with AD. Therefore, progesterone may function as a disease-modifying factor rather than a primary pathogenic trigger [90,91].

Within this framework, a key testable hypothesis is whether within-person increases in progesterone during the luteal phase among women with AD are temporally associated with reduced abundance or metabolic activity of SCFA-producing taxa, lower SCFA concentrations, impaired epithelial barrier function, and greater disease activity. This relationship has not yet been directly demonstrated and should be investigated through longitudinal studies integrating hormonal, microbial, metabolomic, barrier-related, and clinical measurements.

In addition to these endocrine–immunological effects, the gut microbiota may contribute to the local transformation of steroid substrates within the intestinal lumen. Certain intestinal bacteria possess hydroxysteroid dehydrogenases, steroid reductases, and other steroid-transforming enzymes capable of modifying progesterone, testosterone, and related metabolites. However, the presence or activity of these enzymes should not be interpreted as direct evidence that microbial metabolism produces clinically meaningful changes in circulating hormone concentrations in humans. Current evidence suggests that these microbial transformations are more likely to modify the local intestinal sterol pool. Whether they exert systemic endocrine effects depends on several additional factors, including luminal substrate availability, intestinal permeability, metabolite absorption, hepatic first-pass metabolism, conjugation and deconjugation reactions, and the efficiency of enterohepatic circulation. Therefore, microbial steroid transformation should not be directly equated with peripheral hormone clearance [92,93,94,95].

Experimental studies indicate that intestinal bacteria can participate in androgen metabolism, with more extensive evidence currently available for testosterone [92,95]. Evidence also supports microbial transformation of progesterone within the gut, although its direct influence on serum progesterone concentrations in humans remains poorly defined [21]. Overall, human studies simultaneously evaluating microbial enzymatic activity, intestinal steroid metabolites, and circulating progesterone or testosterone concentrations remain extremely limited. Accordingly, the gut microbiota should primarily be considered a local modulator of intestinal steroid metabolism, whereas its potential systemic endocrine effects remain uncertain and depend on intestinal absorption, hepatic processing, and enterohepatic circulation [71,81,93,94,95].

The potential consequences of local microbial steroid transformation may represent a biologically plausible, but still unconfirmed, component of the gut–skin axis in AD. Although changes in the intestinal sterol environment may influence mucosal immune signaling, direct evidence linking microbial steroid transformation to circulating hormone concentrations, cutaneous immune responses, or AD severity is currently lacking [64,81,82]. Reduced systemic androgenic signaling might impair immunoregulatory mechanisms that normally restrict type 2 inflammatory responses, whereas increased progestogenic signaling might enhance these responses in susceptible individuals [9]. However, whether gut microbial steroid metabolism contributes to these systemic endocrine states has not been established. Therefore, its contribution to endocrine–immune balance, skin inflammation, and AD severity should be regarded as a testable hypothesis requiring direct clinical investigation [81,82].

Several limitations should be considered when interpreting the evidence summarized in this review. Much of the available data derives from animal models, pregnancy-associated conditions, in vitro studies, or observational human cohorts. Furthermore, relatively few studies have simultaneously evaluated sex hormones, gut microbiota, skin microbiota, immune biomarkers, and AD outcomes within the same population. Moreover, the literature reviewed did not provide direct longitudinal evidence simultaneously linking menstrual-cycle progesterone fluctuations, SCFA-producing microbial communities or their metabolites, epithelial barrier alterations, and AD severity within the same participants. Consequently, interpretation of the endocrine–microbial axis in AD remains limited by the scarcity of integrative studies simultaneously evaluating hormonal, microbial, immunological, and clinical parameters.

**Figure 1:**
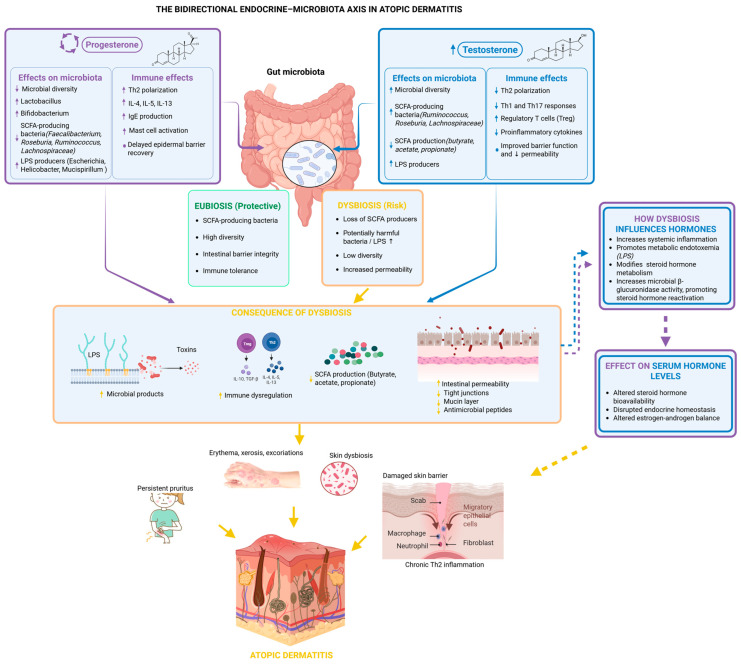
Proposed mechanistic interactions between sex hormones, gut microbiota, immune regulation, and atopic dermatitis (AD). Progesterone and testosterone differentially influence microbial composition, SCFA-producing bacteria, immune responses, and epithelial barrier integrity. Dysbiosis-associated alterations, including reduced microbial diversity, loss of SCFA producers, increased lipopolysaccharide (LPS)-related signaling, and impaired intestinal permeability, may promote Th2-skewed inflammation and epithelial dysfunction. In parallel, gut microbiota may modulate steroid hormone metabolism and bioavailability through mechanisms involving microbial β-glucuronidase activity and steroid transformation pathways. Collectively, these interconnected endocrine–microbial–immune interactions may contribute to the development and progression of AD through the gut–skin axis. The arrows represent proposed biologically plausible relationships and do not indicate experimentally confirmed causal pathways in patients with AD. Note: ↑ indicates an increase; ↓ indicates a decrease.

Collectively, the evidence analyzed in this review suggests that endocrine signaling, microbial metabolism, epithelial barrier integrity, and immune regulation converge within a highly interconnected endocrine–microbial–immune network involved in AD pathophysiology. The mechanisms summarized in Table 5 and illustrated in Figure 1 provide a conceptual framework through which hormonal fluctuations and intestinal dysbiosis may contribute to inflammatory responses, barrier dysfunction, and disease susceptibility in AD. Importantly, these interactions do not appear to follow a uniform biological pattern, but rather depend on the hormonal, microbial, immunological, and metabolic context of each individual. This context dependency may partially explain the remarkable heterogeneity observed in AD severity, recurrence, and therapeutic response across patients. Therefore, future research should focus on integrative and longitudinal methods that can simultaneously assess endocrine profiles, microbial dynamics, immune biomarkers, and epithelial changes. This comprehensive approach could clarify the mechanistic significance and clinical implications of the endocrine-microbial axis in AD.

## 6. Future Perspectives

Current research on the microbiota–hormone–atopic dermatitis axis remains fragmented, as most studies evaluate only one or two components simultaneously, typically microbiota and hormones, microbiota and dermatitis, or hormones and dermatitis. Future investigations should move toward integrative experimental and clinical models capable of evaluating endocrine, microbial, immunological, metabolic, and barrier-related variables within the same framework.

In this context, multi-omic approaches will likely become essential. Metagenomics may provide deeper characterization of microbial taxa, functional genes, and steroid-metabolizing pathways, while metabolomics could identify microbial-derived metabolites, including short-chain fatty acids, bile acids, and tryptophan-related compounds potentially involved in immune regulation and epithelial homeostasis. In parallel, proteomics and transcriptomics may help clarify how microbial and hormonal signals influence inflammatory pathways, epithelial integrity, and immune-cell activation in atopic dermatitis.

A recent study used metagenomics to characterize gut microbial profiles in patients with atopic dermatitis, illustrating the methodological direction of the field [73]. However, future studies should expand on these approaches by incorporating longitudinal hormonal monitoring during major endocrine transitions, including puberty, menstrual cycles, pregnancy, and menopause. Integrating microbial, hormonal, immunological, and clinical data may ultimately enable the development of predictive models capable of characterizing the hormone–microbiota–dermatitis axis with greater precision.

Mendelian randomization studies have provided valuable insights into causal relationships [74]; however, they do not capture the dynamic nature of hormonal and microbial interactions. Longitudinal cohort studies are therefore needed to follow individuals across hormonal transitions while simultaneously assessing microbiota composition, microbial metabolites, immune parameters, epithelial barrier function, and AD progression.

Among these transitions, the menstrual cycle represents a particularly relevant model for investigating short-term hormone–microbiota interactions. Future studies should follow menstruating women with AD across several cycles and compare the follicular and luteal phases through repeated measurements of circulating progesterone, gut microbiota composition, fecal SCFA concentrations, intestinal and skin barrier markers, and AD severity. These analyses should account for potential confounding factors, including diet, hormonal contraceptive use, menstrual-cycle regularity, antibiotic or probiotic exposure, and topical or systemic AD treatments.

Another critical window is the perinatal and early postnatal period, during which maternal hormonal conditions and early microbial colonization may jointly influence immune development and subsequent susceptibility to AD [77]. Maternal progesterone dynamics, mode of delivery, antibiotic exposure, breastfeeding, formula feeding, and complementary feeding may collectively shape the infant microbiota. Prospective mother–infant birth cohorts should therefore evaluate these factors together to determine whether this developmental period may represent a promising entry point for future preventive strategies integrating hormonal, nutritional, and microbiota-related factors.

Therapeutically, some evidence suggests that probiotics containing Bifidobacterium species may contribute to dermatitis prevention in infants, although adult data remain limited and are rarely stratified by sex or hormonal status [74]. Future interventions should consider hormonal status as a potential source of interindividual variability, since microbial responses may differ according to sex, age, and reproductive stage. Ultimately, precision medicine approaches may benefit from integrating both hormonal and microbial profiles when designing preventive and therapeutic strategies for AD.

Beyond probiotics, emerging strategies may include dietary bioactive compounds and personalized microbiota restoration approaches. Preclinical evidence suggests that bioactive polysaccharides can modulate gut microbial composition and contribute to the regulation of glycolipid metabolism [96]. However, whether these microbiota-mediated metabolic effects can modulate inflammatory pathways or provide clinical benefits in AD remains to be established. Although fecal microbiota transplantation has demonstrated therapeutic potential in several inflammatory and infectious disorders, its role in AD remains largely unexplored and requires rigorous clinical evaluation before therapeutic implementation.

Ultimately, the future of atopic dermatitis research may not rely exclusively on suppressing inflammation after disease onset but rather on reconstructing the microbial and hormonal conditions that maintain immune homeostasis before chronic inflammation emerges.

## 7. Conclusions

The evidence reviewed in this study is consistent with a biologically plausible and hypothesis-generating model in which reciprocal interactions between sex hormones and the gut microbiota may modulate gut–skin communication and contribute to AD pathophysiology. Although this integrated model has not yet been established as a causal pathway in patients with AD, understanding these systemic interactions may provide a basis for identifying novel biomarkers, improving patient stratification, and developing personalized preventive and therapeutic strategies. Future advances in AD research will require integrated approaches capable of simultaneously evaluating the endocrine, microbial, immune, metabolic, and epithelial mechanisms underlying disease heterogeneity.

## Figures and Tables

**Table 1:** Summary of the current evidence supporting the role of sex hormones in atopic dermatitis.

Hormone	Evidence Available	Main Populations/Models	Main Outcomes Evaluated	Overall Evidence	Key References
Progesterone	Clinical observational studies, retrospective case series, mechanistic and experimental studies	Infants with AD, women with AD across the menstrual cycle, women with autoimmune progesterone dermatitis, pregnant women, pregnant mouse models, and human immune cell models.	Disease activity across hormonal fluctuations, transepidermal water loss (TEWL), Th2 polarization, PIBF-mediated *STAT6* activation, epithelial barrier function, and progesterone hypersensitivity–associated skin inflammation.	Current evidence suggests that progesterone contributes to type 2 immune polarization and impaired epidermal barrier homeostasis. Clinical studies associate physiological hormonal fluctuations with changes in disease activity, whereas mechanistic and experimental studies support PIBF-mediated immune regulation and progesterone-dependent alterations in epidermal barrier function. However, direct mechanistic evidence in patients with AD remains limited.	[17,19,21,25,26]
Testosterone	Clinical observational, translational, and mechanistic experimental studies	Adult men with AD, human sebocyte cell lines (SZ95 and SEB-1), lesional and non-lesional skin biopsies from patients with AD, transcriptomic datasets from patients with AD, and mouse models of AD.	Serum testosterone levels, disease severity, Th1/Th2/Treg immune responses, HSD3B1 expression, IL-4/IL-13 signaling, local androgen synthesis, lipid metabolism, and epidermal barrier function.	Current evidence suggests that reduced circulating testosterone may be associated with increased disease severity in men with AD. Mechanistic and translational studies further demonstrate that type 2 cytokines regulate local androgen synthesis through *HSD3B1*, which may contribute to lipid abnormalities and epidermal barrier dysfunction. Nevertheless, the relationship between systemic androgen deficiency and local androgen metabolism requires further clinical validation.	[16,39,40]

**Table 2:** Effects of progesterone on gut microbiota composition and diversity. Studies evaluating progesterone-associated microbial alterations across human, animal, and in vitro models.

Hormonal Context	Model	Condition	Microbiota Pattern	Key Taxa	Proposed Biological Implication	Applicability to the AD Context ^1^	Reference
High progesterone (P4) levels during pregnancy	Animal (mouse)	Pregnancy-associated immune thrombocytopenia (PITP)	Decreased α-diversity, with enrichment of potentially inflammation-associated taxa and depletion of bacteria linked to intestinal homeostasis	↑ *Lactobacillus* ↑ *Helicobacter* ↑ *Mucispirillum* ↑ *Turicibacter* ↓ *Blautia* ↓ *Dubosiella* ↓ *Bacteroides* ↓ *Parasutterella* ↓ *Faecalibacterium*	Progesterone-associated microbial remodeling contributed to senescence, apoptosis, and dysfunction of bone marrow mesenchymal stem cells, potentially promoting thrombocytopenia	Indirect but mechanistically relevant SCFA-associated microbial depletion is relevant to AD, but the taxonomic pattern is not directly extrapolable	[48]
High progesterone during pregnancy	Human (pregnant women) and animal (mouse)	Women in the third trimester and mice in late gestation	Progesterone-associated microbial remodeling, particularly Bifidobacterium enrichment	Women: ↑ *Neisseria* ↑ *Blautia* ↑ *Collinsella* ↑ *Bifidobacterium* ↓ *Clostridium* ↓ *Dehalobacterium* ↓ Bacteroidales Mice: ↑ *Anaeroplasma* ↑ *Bifidobacterium*	Progesterone promotes *Bifidobacterium*-enriched communities that may contribute to metabolic and immune adaptations during pregnancy	Indirect but mechanistically relevant *Bifidobacterium* enrichment may support immune homeostasis relevant to AD, but pregnancy-associated microbial changes are not directly extrapolable	[21]
Progesterone exposure	In vitro (clinical *H. pylori* isolates)	Isolates from patients with gastric cancer or duodenal ulcers	Direct bactericidal effect	↓ *H. pylori*	Progesterone directly reduces *H. pylori* viability at high concentrations	Limited applicability Direct inhibition of *H. pylori* was demonstrated, but the finding is not directly extrapolable to AD-associated microbiota	[49]
Endogenous progesterone fluctuations across the menstrual cycle	Non-human primate (*Macaca fascicularis*)	Longitudinal vaginal and rectal sampling across several menstrual cycles	Hormone-associated changes in relative bacterial abundance	↑/↓ *Bacteroides* ↑/↓ *Prevotella* ↑ *Lactobacillus* (in one animal) ↑ *Peptoniphilus* ↓ *Fusobacterium* ↑/↓ *Peptostreptococcus* ↓ Peptostreptococcaceae (individual-dependent association)	Progesterone fluctuations may contribute to cycle-dependent remodeling of vaginal and rectal microbial communities	Limited applicability Hormone-associated microbial shifts were observed, but their relevance to AD-associated gut microbiota is unclear	[50]
Progesterone exposure	In vitro bacterial culture	Direct exposure of selected gut bacteria to progesterone	Changes in bacterial growth and adherence	Growth: ↑ *B. fragilis* ↑ *E. coli* ↑ *L. reuteri* ↓ *B. longum* Adherence: ↓ *E. coli* ↓ *B. longum* ↑ *L. reuteri*	Progesterone can directly modify bacterial growth and adherence independently of host-mediated effects	Limited applicability Direct bacterial responses were demonstrated, but the findings are not directly extrapolable to AD-associated microbiota	[51]
Low versus high progesterone levels	Animal (mouse)	Ovariectomized mice without progesterone replacement versus progesterone supplementation	High progesterone was associated with enrichment of potentially beneficial taxa, whereas low progesterone favored taxa linked to microbial imbalance	High P4: ↑ *L. reuteri* ↑ *L. animalis* ↑ *L. intestinalis* ↑ *Ruminococcus lactaris* Low P4: ↑ *Dorea longicatena* ↑ *Bacteroides stercoris* ↑ *Mucispirillum schaedleri*	Progesterone-dependent compositional shift, with enrichment of *Lactobacillus* spp. after progesterone supplementation and distinct microbial enrichment following ovariectomy.	Indirect but mechanistically relevant *Lactobacillus* enrichment may support immune homeostasis relevant to AD, but the taxonomic pattern is not directly extrapolable	[46]
Gut microbial metabolism of progesterone	Human fecal samples, bacterial isolates, and animal model (female mice)	Women with infertility receiving oral progesterone; experimental administration of progesterone-metabolizing bacteria to mice	Higher abundance and activity of progesterone-metabolizing bacteria; bacterial colonization reduced circulating progesterone in mice	*Clostridium innocuum*	*C. innocuum* converts progesterone into epipregnanolone, reducing progestogenic activity and circulating progesterone availability.	Indirect but mechanistically relevant Microbial progesterone inactivation supports the proposed hormone–microbiota axis, but *C. innocuum* and this pathway have not been evaluated in AD.	[52]

^1^ Applicability indicates the extent to which the reported microbiota changes may be extrapolated to the AD context. This classification does not represent an assessment of methodological quality or risk of bias. No study included in this table directly evaluated progesterone–microbiota interactions in AD. ↑ Indicates increased abundance; ↓ Indicates decreased abundance.

**Table 3:** Effects of testosterone on gut microbiota composition and diversity. Studies evaluating testosterone-associated microbial changes linked to inflammation, microbial diversity, and immune regulation.

Hormonal Context	Model	Condition	Microbiota Pattern	Key Taxa	Proposed Biological Implication	Applicability to the AD Context	Reference
Circulating testosterone and estradiol levels	Human (men and women)	Sex- and hormone-associated differences in gut microbiota	*Paraprevotella* abundance was positively associated with testosterone and negatively associated with estradiol	Men: ↑ *Paraprevotella* Women: ↓ *Paraprevotella*	Sex hormone levels are associated with sex-specific differences in gut microbiota composition	Limited applicability Hormone-associated variation in *Paraprevotella* was identified, but no AD-related microbial mechanism or outcome was assessed.	[59]
Microbiota-induced testosterone elevation	Animal (NOD mouse)	Oral transfer of cecal microbiota from adult male NOD mice to female NOD weanlings predisposed to autoimmune type 1 diabetes	Stable shift to a distinct microbial community, accompanied by increased circulating testosterone	↑ *Roseburia* ↑ *Blautia* ↑ *Coprococcus* ↓ *Peptococcus*	Male microbiota transfer increased testosterone and induced androgen receptor-dependent metabolic and immune changes that protected against autoimmune diabetes	Indirect but mechanistically relevant Testosterone-dependent immune regulation may be relevant to AD, but the microbial pattern and outcomes are not directly extrapolable from an autoimmune diabetes model	[60]
Circulating testosterone and estradiol levels	Human (healthy men and women)	Participants stratified by circulating sex hormone levels (low, medium, and high)	Higher sex hormone levels were associated with greater gut microbial diversity and sex-specific compositional shifts	Men: ↑ *Acinetobacter* ↑ *Dorea* ↑ *Ruminococcus* ↑ *Megamonas* ↓ *Atopobium* Women: ↑ Bacteroidetes ↓ Firmicutes ↑ *Veillonella* ↓ *Slackia* ↓ *Butyricimonas*	Circulating sex hormone levels are associated with sex-specific differences in gut microbiota diversity and composition	Indirect but mechanistically relevant Testosterone-associated enrichment of Ruminococcus, an SCFA-linked taxon relevant to AD, may be biologically meaningful, but the overall microbial pattern is not directly extrapolable.	[61]
Circulating testosterone levels	Human (Elderly Japanese men)	High vs. low testosterone groups	Increased relative abundance of Firmicutes in individuals with higher testosterone levels, with no significant changes in overall diversity	↑ *Alloscardovia* ↑ Clostridiales Firmicutes: ↑ *Turicibacter* ↑ *Gemella*	Specific bacterial taxa may enhance testosterone levels through metabolic processes such as deconjugation and reabsorption, suggesting microbiota-driven endocrine regulation independent of host metabolic factors	Indirect but mechanistically relevant Firmicutes-associated microbial changes may contribute to immune homeostasis relevant to AD, but the findings were obtained in healthy elderly men rather than patients with AD.	[62]
Testosterone and sex modify the composition of gut bacteria	Animal (mouse)	Male vs. female rats (juvenile and adult)	The male gut microbiota increases testosterone more than the female gut microbiota	Men: ↑ *Staphylococcus* ↑ *Jeotgalicoccus* ↑ *Acetanaerobacterium* ↑ *Sporobacter* ↑ *Turicibacter* ↑ *Brevibacterium* Women: ↓ *Lactobacillus* ↓ *Akkermansia*	Testosterone acts on genes that regulate bile acids (increasing FXR, SHP, and ASBT, and decreasing CYP7A1), thereby affecting their composition. By doing so, it modifies the gut microbiota in each individual. This mechanism is called the ‘testosterone-bile acid-microbiota axis’.	Indirect but mechanistically relevant *Lactobacillus* and *Akkermansia* are relevant to epithelial barrier integrity and immune homeostasis in AD, but the microbial changes were identified in animal models and are not directly extrapolable.	[63]
Sex hormone-associated microbial variation	Human (men and women)	Healthy women and men, women with polycystic ovary syndrome	In healthy women, high estrogen levels were associated with a higher abundance of Bacteroidetes compared to Firmicutes (B > F). In healthy men, testosterone levels positively correlated with *Ruminococcus* and Acinetobacter.	Healthy men (high testosterone): ↑ *Ruminococcus* ↑ *Acinetobacter* ↑ Dorea ↑ *Megamonas* ↑ *Raoultella* ↑ *Paraprevotella* Women with POS (high testosterone): ↑ *Escherichia*/*Shigella* ↑ *Streptococcus* ↑ *Clostridium* XIVa ↑ *Rothia* ↑ *Prevotella*_9 ↑ *Ruminococcus*_2 ↑ *Collinsella* ↓ *Akkermansia* ↓ *Coprococcus* ↓ *Ruminococcus*	It is postulated that an individual’s microbiota can metabolize sex hormones using enzymes such as β-glucuronidase, while hormone levels also influence both the quantity and diversity of the microbiota.	Indirect but mechanistically relevant Hormone-associated changes in *Akkermansia*, *Coprococcus*, and *Ruminococcus* may influence microbial homeostasis relevant to AD, but the findings were obtained in healthy individuals and women with polycystic ovary syndrome rather than patients with AD.	[64]
Sex hormone–microbiota associations across adulthood	Human observational studies and experimental bacterial evidence	Healthy adults, menopausal status, and prostate cancer populations	In adulthood, there are significant differences in the gut microbiota between women and men, and this microbial variation can influence circulating levels of testosterone and progesterone.	Testosterone-associated: ↑ *Acinetobacter* ↑ *Dorea* ↑ *Megamonas* ↑ *Ruminococcus* ↑ Fibrobacteriaceae ↑ Idiominaceae ↓ Verrucomicrobia ↓ Akkermansiaceae	Experimental evidence indicates that bacterial 3β-HSD activity can transform testosterone into downstream steroid metabolites. However, this enzymatic capacity should not be interpreted as direct evidence of peripheral testosterone clearance or clinically meaningful reductions in circulating testosterone concentrations in humans.	Indirect but mechanistically relevant demonstrates bacterial testosterone transformation, but no AD-specific or direct systemic endocrine effects were evaluated.	[65]
Testosterone levels (physiological vs. exogenous)	Animal (mouse)	Normal vs. orchiectomy (ORX) vs. testosterone propionate (TP); healthy vs. tumor model	Under physiological conditions, testosterone is associated with enrichment of Firmicutes and butyrate-producing taxa, whereas testosterone depletion (ORX) reduces these beneficial bacteria. In contrast, exogenous testosterone under pathological conditions promotes opportunistic taxa and reduces commensals.	↑ Firmicutes ↑ Lachnospiraceae butyrate producers: ↑ *Flintibacter butyricus* ↑ *Ruminococcus bromii* ↑ *Kineothrix alysoides* ORX: ↓ *Ruminococcus bromii* ↓ *Flintibacter butyricus* TP (tumor model): ↑ *Akkermansia muciniphila* ↑ *Mucispirillum schaedleri*	Testosterone exerts context-dependent effects on microbiota, promoting beneficial butyrate-producing taxa under physiological conditions but favoring dysbiosis under pathological states	Indirect but mechanistically relevant Enrichment of butyrate-producing taxa and SCFA-associated bacteria supports epithelial barrier integrity and immune homeostasis relevant to AD. However, these findings were obtained in mouse models rather than in AD.	[47]
Normal/high testosterone levels	Animal (mouse)	Male-associated microbiota	Enrichment of androgen-associated microbial taxa	↑ Actinobacteria ↑ Tenericutes ↑ *Allobaculum* ↑ *Anaeroplasma* ↑ *Erwinia* ↑ *Akkermansia* ↑ *Sutterella*	Testosterone promotes the expansion of specific microbial taxa potentially linked to metabolic and barrier functions	Indirect but mechanistically relevant Expansion of *Akkermansia* and other androgen-associated taxa may support epithelial barrier and metabolic homeostasis relevant to AD, but the taxonomic pattern is not directly extrapolable.	[66]
Low testosterone (GDX)	Animal (mouse)	Hormone depletion	Shift toward altered microbial composition with enrichment of specific taxa	↑ Ruminococcaceae	Loss of testosterone favors expansion of taxa associated with dysbiosis and altered metabolic states	Limited applicability Hormone-associated microbial changes were observed, but their relevance to AD-associated gut microbiota is unclear.	
Sex hormone–associated microbial correlations	Animal (mouse)	Sex-based hormonal differences (E2 vs. testosterone)	Positive correlation with estradiol and negative correlation with testosterone for specific taxa	↑ with estradiol and ↓ testosterone: Muribaculaceae *Turicibacter* *Parasutterella* ↑ *with testosterone:* *Bifidobacterium* Gammaproteobacteria *Enterococcus* *Streptococcus*	Sex hormones selectively enrich or suppress specific microbial taxa, indicating hormone-dependent ecological niches within the gut microbiota	Indirect but mechanistically relevant *Bifidobacterium* enrichment may support immune homeostasis relevant to AD, but the hormone-associated microbial changes are not directly extrapolable.	[67]
Physiological increase in testosterone (puberty)	Animal (mouse)	Pre- vs. post-puberty	No differences before puberty; clear divergence in microbial composition after puberty	Clostridiales (↑ in males)	Sexual maturation drives microbiota differentiation, highlighting hormones as key determinants of microbial structure	Limited applicability Hormone-associated microbial changes were observed during puberty, but their relevance to AD-associated gut microbiota is unclear.	[68]
Sex-dependent hormonal environment	Animal (mouse)	Males vs. females (post-puberty)	Distinct sex-specific microbial profiles emerge after puberty	*Turicibacter,* Enterobacteriaceae: ↑ in females No changes (in males) ↑ Bacteroidetes ↓ Proteobacteria	Hormonal differences between sexes shape microbiota composition linked to metabolic and inflammatory traits	Limited applicability Sex-dependent microbial differences were observed, but their relevance to AD-associated gut microbiota is unclear.	
Direct exposure to testosterone/DHT	In vitro	Pre-pubertal fecal samples	Hormone-sensitive microbiota shifts in early developmental stage		Early-life microbiota exhibits increased hormonal sensitivity, suggesting critical windows of microbial plasticity	Indirect but mechanistically relevant Hormone-induced shifts in major bacterial phyla may influence microbial homeostasis relevant to AD, but the findings were obtained in vitro and are not directly extrapolable.	
Exogenous testosterone (acute exposure)	Animal (mouse)	Injection (3 days)	Rapid disruption of microbial balance in females	↓ *Bifidobacterium*	Acute hormonal changes can reduce beneficial taxa involved in immune regulation	Indirect but mechanistically relevant *Bifidobacterium* depletion may impair immune homeostasis relevant to AD, but the findings are not directly extrapolable.	

Note: ↑ Indicates increased abundance; ↓ Indicates decreased abundance.

**Table 4:** Gut microbiota alterations in atopic dermatitis. Studies evaluating dysbiosis-associated microbial patterns, SCFA-producing bacteria depletion, and inflammatory-related microbial changes in AD.

Population/Cohort	Microbiota Pattern	Key Taxa	Mechanistic Axis	Biological Implication	Reference
Children (0–6 years)	Reduced microbial diversity with loss of SCFA-producing bacteria and enrichment of pro-inflammatory taxa	SCFA producers ↓ (*Bifidobacterium*, *Blautia*, *Coprococcus*); *Bacteroides* ↑, *Sutterella* ↑	SCFA depletion/Th2 inflammation	Reduced SCFA production and increased LPS signaling may promote intestinal permeability and Th2-skewed inflammation	[72]
Multi-cohort (MiBioGen + FinnGen)	Specific microbial taxa associated with increased or decreased risk of AD	Risk-associated ↑: *Bacteroides*, Clostridiaceae, *Anaerotruncus*. Protective ↑: *Bifidobacterium*, *Lactobacillus*, Christensenellaceae, *Anaerostipes*	Immune regulation/SCFA metabolism	Microbial composition may influence AD susceptibility through modulation of Treg/Th1/Th2 balance and SCFA-related pathways	[74]
Adults (Hong Kong, China)	Dysbiosis associated with disease severity and reduced SCFA-producing taxa	*Blautia* ↑, *Butyricicoccus* ↑, *Romboutsia* ↓, ↑ Bacteroides	Functional metabolic dysbiosis	Reduced abundance of SCFA-producing taxa and altered microbial metabolic pathways may contribute to impaired epithelial integrity, Th2-skewed inflammation, and gut–skin axis dysfunction.	[75]
Adults (Spain)	Reduced microbial diversity and impaired functional capacity	Ruminococcaceae ↓, *Akkermansia muciniphila* ↓; Bacteroidaceae ↑, *Escherichia coli* ↑	Barrier dysfunction/metabolic dysbiosis	Loss of beneficial taxa may impair epithelial integrity and immune regulation	[73]
Adults and infants (Seoul, Korea)	No major changes in overall diversity, but dysbiosis at sub-species and functional level with reduced SCFA production Functional dysbiosis with reduced SCFA production despite limited diversity changes	*Faecalibacterium prausnitzii*—low butyrate-producing	SCFA depletion	Reduced butyrate production may increase intestinal permeability and sustain Th2-mediated inflammation	[76]
Infants (early-life microbiota)	Reduced microbial diversity with delayed colonization of beneficial taxa and enrichment of pathogenic bacteria	*Escherichia coli* ↑, *Klebsiella pneumoniae* ↑, *Clostridium difficile* ↑; *Bifidobacterium* ↓	Immune maturation defect	Early dysbiosis may impair immune maturation, promoting Th2 responses and increasing risk of AD development	[77]
Infants (Netherlands)	Colonization by *C. difficile* at 1 month is associated with increased risk	*C. difficile* ↑	Increased intestinal permeability	*C. difficile* can increase intestinal permeability induced by its toxins	[78]
Japanese infants (6 months)	Relationship between *Bifidobacterium* and atopic dermatitis	1-month: *Bifidobacterium catenulatum* ↑, *B. adolescentis* ↑. 3-month: *Bifidobacterium catenulatum* ↓, *B. breve* ↑. 6-month: *B. bifidum* ↑	Early immune programming.	Risk between Bifidobacterium and allergies.	[79]
Pregnant women and infants of 1 week, 1 month and 1 year.	Altered microbial diversity associated with eczema susceptibility	Pregnant women: Bacteroidetes ↓. Infants: Ruminococcaceae ↓, Enterobacteriaceae ↑	Inflammatory signaling	Low abundance of taxa was associated with high levels of inflammatory cytokines	[80]

Note: ↑ Indicates increased abundance; ↓ Indicates decreased abundance.

**Table 5:** Proposed endocrine–microbial mechanisms in atopic dermatitis. Mechanistic pathways linking sex hormones, gut microbiota alterations, immune dysregulation, and epithelial barrier dysfunction in AD.

Mechanistic Category	Representative Microbial Alterations	Representative Studies	Integrated Endocrine-Microbiota-AD Mechanism
SCFA depletion and immune dysregulation	↓ *Bifidobacterium* ↓ *Blautia* ↓ *Coprococcus* ↓ *Faecalibacterium prausnitzii* ↓ Ruminococcaceae	[72]	Loss of SCFA-producing and immunoregulatory taxa may impair epithelial homeostasis, reduce immune tolerance, and favor Th2-skewed inflammatory responses associated with AD.
Barrier dysfunction and intestinal permeability	↑ *Escherichia coli* ↑ *Clostridium difficile* ↑ Bacteroidaceae ↓ *Akkermansia muciniphila*	[72]	Expansion of pro-inflammatory or toxin-producing bacteria together with depletion of barrier-supportive taxa may increase intestinal permeability and promote systemic inflammatory signaling relevant to AD.
Early-life microbial programming and allergy susceptibility	Delayed colonization by *Bifidobacterium* spp. ↑ *Klebsiella pneumoniae* ↑ Enterobacteriaceae	[77,79,80]	Early-life dysbiosis may impair immune maturation and microbial tolerance, favoring allergic sensitization and Th2-skewed responses associated with increased AD susceptibility. Maternal progesterone dynamics, mode of delivery, and feeding practices may jointly influence infant microbial colonization. This developmental period may therefore represent a promising window for future studies integrating hormonal, nutritional, and microbiota-related preventive strategies.

Note: The mechanisms summarized in this table represent hypothesis-generating integrations derived largely from indirect human, animal, and experimental evidence. They should not be interpreted as confirmed causal pathways in patients with AD. ↑ Indicates increased abundance; ↓ Indicates decreased abundance.

## Data Availability

No new data were created or analyzed in this study. Data sharing is not applicable to this article.
